# Impact of left vs. right hemisphere stroke on driving: lateralized attention deficits and executive dysfunction linked to impaired driving

**DOI:** 10.3389/fstro.2026.1679668

**Published:** 2026-03-18

**Authors:** Krista Schendel, Isabella Santavicca, Timothy J. Herron, Sandy J. Lwi, Brian C. Curran, Jas M. Chok, Juliana Baldo

**Affiliations:** 1 VA Northern California Health Care System, Martinez, CA, United States; 2 Department of Neurology, University of California, Davis, Davis, CA, United States

**Keywords:** aphasia, automobile driving, executive function, hemispheric differences/laterality, lateralized attentional deficit, stroke, visuospatial attention

## Abstract

**Introduction:**

For many people, driving is essential to quality of life because it facilitates social integration and community participation. Indeed, many stroke survivors return to driving within months post-stroke. Few studies, however, have specifically characterized post-stroke driving errors as a function of affected hemisphere (LH vs. RH) and cognitive impairment.

**Methods:**

This study examined driving performance in LH and RH stroke survivors and age-matched controls using a fully interactive driving simulator.

**Results:**

Analysis revealed that the direction and severity of visuospatial attention deficits were significant predictors of post-stroke driving performance and executive dysfunction correlated with specific types of driving errors. Moreover, the cerebral hemisphere affected by stroke had a significant impact on lane positioning errors, with RH stroke survivors experiencing more difficulty maintaining lane position. In addition, a higher incidence of lane departures on the contralesional side of the lane was observed after stroke. Notably, neither age, months post-stroke, nor simple reaction time were reliably associated with scores or pass/fail ratings on the simulated driving assessment.

**Discussion:**

This work highlights how LH and RH strokes differentially impact driving and suggests that driving assessment and rehabilitation efforts should consider both the direction and severity of visuospatial attention deficits as well as the degree of executive dysfunction in stroke survivors who wish to continue driving.

## Introduction

Stroke is a leading cause of disability in the United States, affecting approximately 800,000 Americans each year (U.S. Centers for Disease Control and Prevention, 2023; Tsao et al., 2023). While overt disabilities such as aphasia and sensorimotor impairments are a consistent focus of rehabilitation, an area that has been relatively underappreciated is the impact of stroke on driving. The ability to drive is often synonymous with independence in our society, so many older adults are reluctant to give up this

privilege even after experiencing a stroke or other brain injury. Despite evidence that brain-injured patients are at increased risk of motor vehicle crashes (Devos et al., 2015; Lundqvist et al., 2000; McGwin et al., 2000; Perrier et al., 2010; Reston et al., 2008; Sims et al., 2012), prior reports have estimated that up to 30–50% of stroke survivors will return to driving within the first year (Aufman et al., 2013; Fisk et al., 1997; Lee et al., 2003; Legh-Smith et al., 1986; Yu et al., 2016) with as many as 25% returning to driving within the first month.

Prior studies have also shown that few stroke patients receive formal driving assessments or retraining prior to returning to driving (Fisk et al., 1997; Yu et al., 2016; Akinwuntan et al., 2012; Petzold et al., 2010). Numerous reasons contribute to this gap in care. First, on-road driving assessments can be costly and time-consuming. Second, stroke rehabilitation often prioritizes language, visual, physical, and cognitive impairments, often leaving fitness to drive unaddressed (Petzold et al., 2010). Moreover, although many U.S. physicians are currently required to report conditions related to lapses of consciousness to the Department of Motor Vehicles (DMV), requirements for reporting other conditions are less clear. In California, for example, physicians are asked to report other disorders related to lapses of consciousness if they are “…severe enough to be likely to impair a person's ability to operate a motor vehicle…” (FindLaw, 2023). Although post-stroke seizures can and do occur, many stroke survivors do not experience seizures or any lapses of consciousness. While patients experiencing seizures are likely to have driving restrictions put in place, many stroke survivors will continue driving without being evaluated for driving safety, underscoring the need for a more comprehensive understanding of how strokes can impact driving.

Several cognitive impairments associated with stroke can negatively impact driving. For example, right hemisphere (RH) brain injury is often associated with significant visuospatial impairments such as hemispatial neglect or extinction causing individuals to fail to notice stimuli located to the left (Deouell et al., 2005; Heilman et al., 2000; Mesulam, 2002; Posner et al., 1984; Ringman et al., 2004; Roy et al., 1987). Recent studies indicate that such RH deficits can adversely affect driving (Shimonaga et al., 2021; Sotokawa et al., 2024), including road positioning (Spreij et al., 2020). Although typically less severe, similar impairments in visuospatial attention have also been documented after left hemisphere (LH) stroke (Beis et al., 2004; Stone et al., 1991) and can persist many years post-stroke (Schendel et al., 2016). Although milder forms of visuospatial inattention are harder to detect with standard paper and pencil tasks (Deouell et al., 2005; Azouvi et al., 2002; Schendel and Robertson, 2002), it has been shown that mild impairments in visuospatial attention can be exacerbated under conditions of multitasking (Blini et al., 2016; Bonato, 2012). Thus, in a driving environment where accurate and timely responses to external stimuli and multitasking are critical, LH stroke survivors (and RH stroke survivors without frank neglect) may be more vulnerable to impairments in visuospatial attention than previously appreciated. Notably, a study by (Park 2015) reported that 50–60% of individuals with LH stroke make serious driving errors, although the role of visuospatial attention was not directly assessed (Park, 2015). Further research with sensitive measures of visuospatial impairment is therefore needed to determine the degree to which impairments in visuospatial attention after LH or RH stroke impact driving (Spreij et al., 2020; Azouvi et al., 2002; Akinwuntan et al., 2010; Schanke and Sundet, 2000).

Another common cognitive impairment that may disrupt driving performance is executive dysfunction. Executive dysfunction can disrupt one's ability to assess a multitude of information and then plan and coordinate a relevant behavioral response. Prior studies have noted the importance of executive functioning abilities in driving, with implications for crash frequency and traffic violations in young adults with ADHD (Barkley et al., 2002) as well as for lane positioning and other errors in healthy older adults (Aksan et al., 2013; Demireva et al., 2012). Another study indicated that executive dysfunction has the greatest impact during challenging driving scenarios and complex situations (Gillen and Rubio, 2011). Previous research has further indicated that the Trails B test, a measure of executive functioning, is one of the best cognitive predictors of on-road driving performance (Devos et al., 2011; Marshall et al., 2007). Other research including older drivers has also established that cognitive functioning plays a distinct role in driving safety, above and beyond that of basic vision (Anderson et al., 2012), particularly executive functioning and visuospatial ability (Aksan et al., 2013). Nonetheless, few studies have directly investigated the impact of impairments in executive functioning and visuospatial attention on driving in post-stroke populations (Motta et al., 2014).

The impact of language impairment on driving has also been debated in the literature. Individuals with aphasia do experience barriers in returning to driving, but it is unclear whether this is due to reduced fitness to drive or an uncertainty among care providers regarding the role of language in driving (see Gasne et al., 2024; Wallace et al., 2024). While some research has demonstrated that aphasia impairs on-road driving (Marshall et al., 2007; Hartje et al., 1991; Nouri et al., 1987), other research has found little or no impact (Berger and Mensh, 2011; Breen et al., 2016, 2017; Breen, 2021; Nouri and Lincoln, 1992). This discrepancy highlights the need to assess the presence of aphasia in relation to driving performance.

Finally, prior research examining the impact of stroke on driving has rarely compared the types of driving errors (e.g., lane positioning, speeding, etc.,) made by individuals with a LH vs. RH stroke. Examining lane departure errors, for example, is important because a tendency to swerve left or right has very different consequences depending on the side of oncoming traffic. Collisions, missed stop signs, and poor speed management are also important indicators of unsafe driving, emphasizing the importance of understanding how such errors may vary as a function of lesion location and cognitive functioning. Given that brain injuries involving the left or right cerebral hemisphere produce very different cognitive challenges, it follows that a highly demanding cognitive task like driving may be uniquely impacted as well. The current study addressed this gap by examining the impact of LH vs. RH stroke and associated cognitive impairments on simulated driving under challenging driving conditions.

This study thus addresses several gaps in the literature by relating driving performance to executive functioning, visuospatial attention, and linguistic ability in both LH and RH stroke survivors. Individuals were tested in a driving simulator that included fully interactive steering, acceleration, and braking which allowed for testing in realistic and complex driving scenarios (Akinwuntan et al., 2012, 2010, 2005; Devos et al., 2010, 2016; Rosen and Wachtel, 2004). Simulators have been previously shown to be highly accurate in predicting on-road driving performance (Akinwuntan et al., 2011; Aksan et al., 2016). Unlike on-road driving tests, however, the driving simulator allowed us to test identical driving scenarios across all participants, including a multitude of challenging situations that do not typically occur in on-road driving tests (e.g., navigating through construction zones, numerous pedestrians, unexpected roadblocks, and multiple speed limit changes). Based on prior literature, we expected executive functioning to be predictive of overall driving performance in all participants. We also predicted that RH and LH stroke survivors would exhibit different patterns of driving errors. Specifically, we hypothesized that RH stroke survivors would exhibit more lane positioning errors and collisions due to spatial inattention, while LH stroke survivors would have difficulty managing speed due to executive dysfunction. We further hypothesized that stroke survivors would exhibit more errors on the contralesional side of the lane.

## Materials and methods

### Participants

In the current study, 71 participants (13 female) from our VA Medical Center were tested on an interactive driving simulator to assess driving performance. This cohort study included Veterans with a history of a single LH (*n* = 28) or RH stroke (*n* = 23), as well as a group of healthy age-matched Veteran controls (*n* = 20, see Table 1 for demographic data). Inclusion criteria for the stroke participants were: History of a single unilateral stroke (at least 3-months post-onset) as confirmed via computerized medical record which included stroke dates and diagnoses based on physical impairments, neurological exam, and radiology reports; aged 40–80; pre-morbidly right-handed based on the Edinburgh Handedness Questionnaire (to minimize unusual lateralization patterns); a minimum of 12 years of education or high school diploma equivalent; premorbid driving history; and English language proficiency. Exclusion criteria included: History of pre-morbid neurologic illness (e.g., additional strokes, TBI, dementia, Parkinson's, etc.); severe psychiatric history (e.g., schizophrenia, bipolar disorder); substance abuse within the past year, and legal blindness. Veterans receiving care within the VA medical system are routinely screened for TBI symptoms, and no participants with a history of TBI with loss of consciousness, positive neuroimaging findings, amnesia, or altered mental status were included (Silverberg et al., 2023). A remote history of concussion, however, was not an exclusion criterion since it is so common among the US population (Daugherty et al., 2023). Stroke survivors were not excluded based on the presence of aphasia or hemiparesis. Those with severe hemiparesis drove with one hand and were allowed to use their non-affected foot for the gas/brake. Visual acuity was not part of the exclusion criteria as long as acuity exceeded the U.S. limit for legal blindness (i.e., 20/200 or better in at least one eye as determined by computerized testing with the Freiburg Tumbling E Visual Acuity Test; Bach, 1996, 2007).

**Table 1:** Table of participant groups (Controls, LH stroke, and RH stroke) showing group means (and standard deviations) for age, education, and grip strength in the right (R) and left (L) hands.

**Group**	***n***	**Age [yrs]**	**Education [yrs]**	**Grip R [kgs]**	**Grip L [kgs]**	**Months post-stroke (MPO)**
Controls	20	69.25 (7.62)	16.05 (2.09)	31.06 (8.72)	28.71 (9.45)	-
LH stroke	28	65.71 (10.52)	14.61 (2.38)	28.74 (13.37)	34.25 (10.41)	109.39 (6–396)
RH stroke	23	69.26 (7.73)	14.96 (2.53)	33.47 (10.53)	27.07 (13.72)	95.83 (3–249)

For the two stroke groups, months post-stroke onset (MPO) is shown as mean (and range).

Age-matched control participants met the same inclusion/exclusion criteria listed above except they had no stroke history and no handedness requirement (two controls were left-handed). Participants were not required to be currently driving, but 78% of stroke patients and all controls were currently driving. A multivariate analysis of variance examining variables of age and education across study groups (Controls, LH stroke, RH stroke) revealed no group differences (both *p* > 0.100).

All participants provided informed consent consistent with the Declaration of Helsinki and the Belmont Report prior to participating in the study. All participants were told that this study was being conducted for research purposes only and should not be considered an official assessment of fitness to drive. The study was approved by and conducted under the direction of the Institutional Review Board (IRB00000615) of the VA Northern California Health Care System (FWA00001687).

### Driving simulation

Driving performance was assessed with the DrivingLab/University of Kansas Medical Center Portable Driving Simulator, which has an interactive steering wheel, gas and brake pedals. The driving simulator operated on STISIM Drive software (STI Inc, Hawthorne, CA), version 3.15.07, with a custom-designed front- and back-end to enhance user-friendliness. It was equipped with a single 22-inch screen, along with a Logitech^®^ steering wheel and pedals (Logitech International S.A., Lausanne, Switzerland).

All participants in the study performed the same pre-programmed driving scenarios. First, participants completed a familiarization drive (approximately 14 min in duration) that allowed participants to practice using the steering wheel, gas and brake pedals, and learn the location of the speedometer, rear and side-view mirrors, and turn signals. Participants were allowed to control the gas and brake pedal with whichever foot they preferred. All participants used their right foot for the gas pedal, and the majority used the right foot for the brake pedal as well. The only exceptions were two stroke survivors (LH), both of whom used their left foot for the brake and their right foot for the gas. Most participants used both hands on the steering wheel, although 3 LH stroke and 4 RH stroke participants used only their ipsilesional hand due to hemiparesis of the contralateral arm.

**Figure 1:**
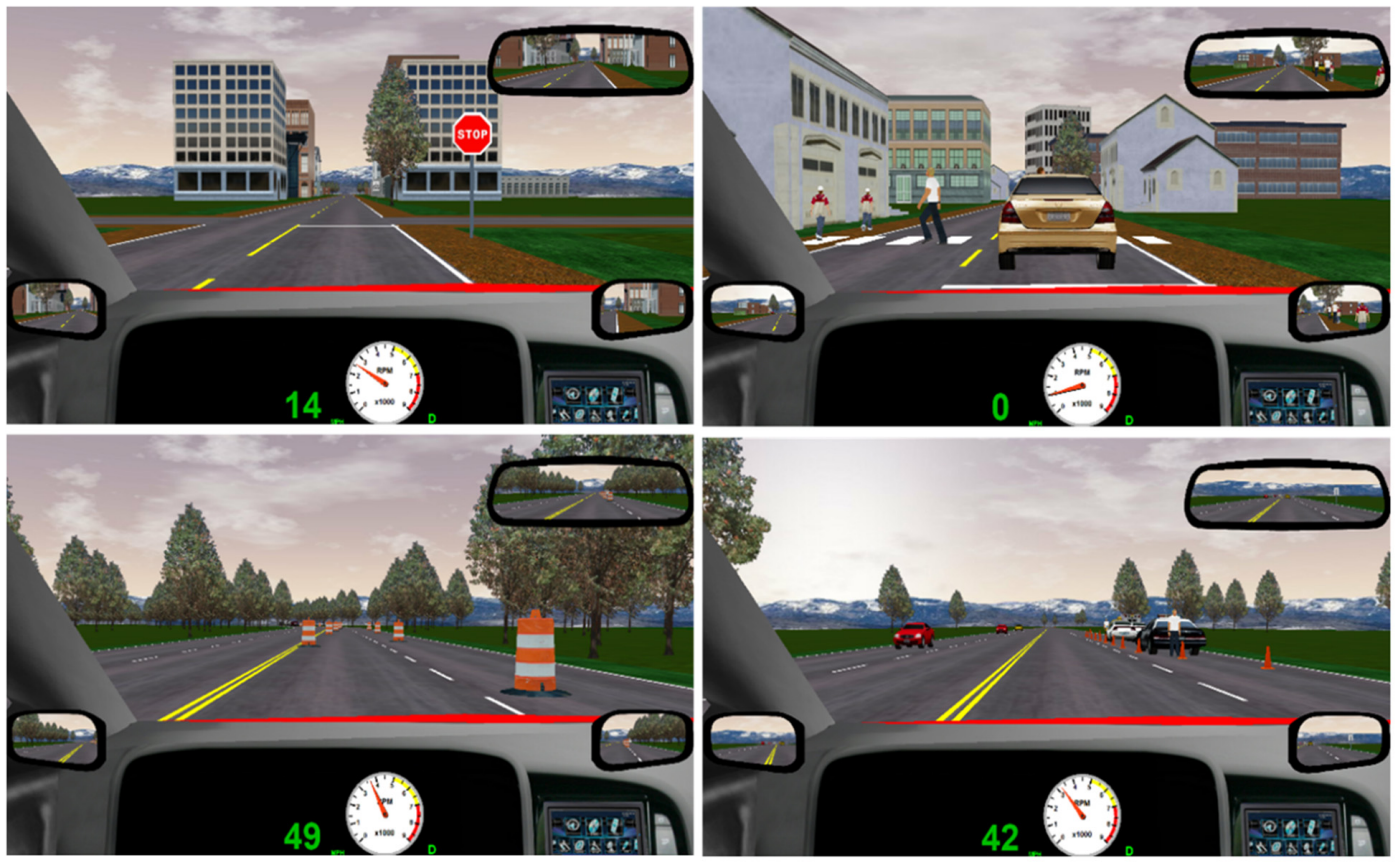
Screenshots of selected scenes from the simulated driving assessment showing a stop sign intersection, a crosswalk with pedestrians, a construction cone zone, and a car blocking part of the roadway.

Next, participants completed the main driving assessment (approximately 15 min). This assessment mimicked real-life driving with several challenges, such as unexpected cars and pedestrians entering the roadway (one from the left and one from the right), oncoming traffic, straight and curving roadways, slower vehicles ahead, and frequent changes in posted speed limits. The assessment drive included realistic graphics of city streets and scenic country roads, including two stop signs, one traffic light, two required turns (one left, one right), two required merge points, six pedestrian crossings, one construction zone, and two vehicles blocking part of the roadway (see Figure 1).

The following driving errors were recorded by the simulator software during the driving assessment and used to calculate the driving assessment score: Speed exceedances, collisions, hit pedestrians, and missed stops. The driving score was determined by calculating a weighted sum of these driving errors, with higher scores indicating more errors. This score also determined the final driver assessment rating, categorized as passing (0–8 points), moderately impaired (9–16 points), or failing (17+ points). Lane positioning errors were also recorded by the driving simulator software, including the number of leftward vs. rightward lane departures as well as the percentage of time spent outside of the lane (TOL), i.e., the total percentage of the time the car was not positioned within the lane lines.

Symptoms of motion sickness were assessed with the Simulator Sickness Questionnaire (Kennedy et al., 1993) which was administered prior to any driving (baseline), after the familiarization drive, and again after completion of the driving assessment. Several preventative measures were also taken to minimize the chance of simulator sickness, including cool room temperature, air flow, extra breaks in an adjacent outdoor space as needed, and the availability of mints and ginger chews. Feelings of motion sickness were easily rectified by taking a break with fresh air and eating a mint or ginger chew. However, one individual from the stroke cohort experienced ongoing discomfort and was unable to complete the driving assessment. Thus, this individual's data were not included.

### Neuropsychological testing

Cognitive testing was conducted on a separate day from the driving assessment, with the order of driving and cognitive testing sessions counterbalanced across participants. All participants completed a neuropsychological battery that included measures of executive functioning, language ability, and visuospatial attention.

Executive functioning was measured with the Symbol Trails task from the Cognitive Linguistic Quick Test (CLQT). This test battery was developed as a non-verbal neuropsychological assessment for individuals with cognitive limitations such as aphasia (Helm-Estabrooks, 2018). Here, we used the Symbol Trails subtest from the CLQT as a measure of executive functioning. The Symbol Trails task differs from the traditional Trails task in that participants are asked to connect items based on shape and size rather than the sequencing of numbers and letters, which can be problematic in individuals with aphasia. The critical third trial of the Symbol Trails task involves connecting items while alternating between shapes (circles and triangles) as they increase in size (e.g., small triangle, small circle, next largest triangle, next largest circle, etc.). The number of errors on this switching trial is used as a measure of executive dysfunction.

Language functioning was assessed in all stroke participants with the Western Aphasia Battery-Revised (WAB; Kertesz, 2007; Shewan and Kertesz, 1980). The WAB is a speech and language battery with subtests that assess speech fluency, naming, repetition, and auditory comprehension. An overall Aphasia Quotient (AQ) is calculated based on a summary of these subtest scores providing a measure of aphasia. Higher WAB AQ scores (up to 100) reflect better speech and language performance, with a score >93.7 considered within normal limits for speech/language status. This WAB cutoff score was thus used to distinguish presence or absence of aphasia.

To test for visuospatial attention deficits, we administered the visual conjunction search task developed by Schendel and colleagues (List et al., 2008), which requires participants to visually search a cluttered display for a target defined by a conjunction of features, namely a red square (color + shape), among a field of distractors that contained one, but not both features (e.g., red triangles, or blue squares). This task requires serial attentional search and is designed to capture lateralized visuospatial attention impairments, such as neglect, in brain injured individuals (Schendel et al., 2016; Oren et al., 2013). In this task, participants are given unlimited time to respond and allowed to respond either verbally or by gesture, making it appropriate to administer to individuals with aphasia or motor impairments. The task's adaptive staircase procedure produces two separate target detection thresholds (based on stimulus duration), one for targets appearing on the left and another for those appearing on the right (see List et al., 2008, for more detail). The difference between each participant's left and right detection thresholds thus provides a metric of attentional imbalance. In order to normalize each participant's difference scores with respect to their individual threshold levels (which ranged between 59 and 1,400 ms), a normalized score of attentional imbalance (AIM score) was calculated for each participant as the difference between their left and right thresholds (L_threshold_–R_threshold_) divided by the sum of the two thresholds (Oren et al., 2013; Behrmann and Tipper, 1999), with positive values representing a bias toward the right (and negative values reflecting a leftward bias).

For stroke participants, stimulus location can also be defined with respect to the injured hemisphere (contralesional vs. ipsilesional). The severity of contralesional attention deficit after stroke was thus calculated as the ratio between each stroke participant's contralesional/ipsilesional target thresholds, with the resulting score indicating how much more viewing time was needed to detect targets located on the contralesional (vs. ipsilesional) side of the display. For example, a contralesional attention deficit score of 2.0 would indicate that the individual required a viewing time that was twice as long for contralesional compared to ipsilesional targets.

In addition to the cognitive tasks, several sensorimotor tasks were also administered: Mean grip strength was computed as the average grip strength between the two hands (measured by hand dynamometer), visual acuity via the Freiburg Visual Acuity test (Bach, 1996), and a simple reaction time task from the California Cognitive Assessment Battery (Woods et al., 2024).

## Data analysis

### Driving errors after stroke

Differences in the types of driving errors committed after LH vs. RH stroke were analyzed via multivariate analysis of variance (MANOVA) with stroke hemisphere (RH, LH) as the independent variable. Lane positioning errors (leftward/rightward lane departures and TOL) were analyzed in one analysis, while errors that directly contributed to the overall driving score (collisions, pedestrians hit, speeding, and missed stops) were analyzed in a separate analysis. Finally, a repeated-measures ANOVA was used to test for an interaction between stroke hemisphere (LH, RH) and direction of lane departures (left, right).

### Driver assessment ratings

Group differences in driver assessment ratings (i.e., passing, moderately impaired, or failing) were analyzed via a single-sided ordinal logistic regression analysis (MASS package, R v. 4.0.2). A linear regression model was used to determine whether differences in ratings could be attributed to demographic (age, education) or sensorimotor factors (grip strength, visual acuity, and simple reaction time). In addition, Spearman rank correlations were used to test the degree to which demographic, sensorimotor, and cognitive factors correlated with the driver assessment ratings.

### Group characteristics

Group differences (RH stroke, LH stroke, and controls) in executive functioning (Cognitive Linguistic Quick Test) and visuospatial attention (conjunction search thresholds and AIM scores) were analyzed with analysis of variance (ANOVA). Group differences in linguistic ability (above or below the WAB cut-off score for presence of aphasia) and severity of contralesional deficit were also analyzed with ANOVA, but only included data from the stroke survivors since these measures were not obtained in the control participants.

### Factors associated with driving performance

Pearson correlation analyses (corr package in R) were conducted to assess the degree to which specific cognitive, sensorimotor, and demographic factors were associated with overall driving score and different types of driving errors. As these were planned analyses, *p*-values were not corrected for familywise error. A hypothesis-driven step-up general linear regression analysis was also conducted to determine which cognitive, linguistic or sensorimotor factors were most predictive of participants' driving scores (i.e., overall driving performance, *n* = 71). In order to reduce the impact of outliers, driving scores were winsorized (±2 standard deviations from the mean). The first regression model included the factors that we hypothesized would be most predictive of driving score, namely: group (LH, RH, Control), attentional imbalance (AIM Score), and executive dysfunction. To test for contributions from sensorimotor factors, the second model added factors of mean grip strength and simple reaction time while dropping any factors that achieved a criterion level of *p* > 0.30 in the prior model, in order to reduce noise and balance the total number of factors against total sample size in subsequent regressions. The final step-up linear regression analysis was restricted to the stroke participants' winsorized driving scores (*n* = 51). All factors from the main analysis that achieved a criterion value of p < 0.30 were included, along with additional stroke-specific factors that were shown to correlate with increased driving errors (e.g., severity of contralesional attention deficit, presence of aphasia, and months post-stroke onset). Effect sizes (η^2^) are reported and interpreted according to Cohen's (1988) guidelines. Finally, general linear regression analyses (R v. 4.0.2) were used to test whether scores on the Simulator Sickness Questionnaires were predictive of driving scores. Since Simulator Sickness scores were distributed with a single tail and skewed toward 0, a gamma distribution model was used.

## Results

### Driving assessment ratings

**Figure 2:**
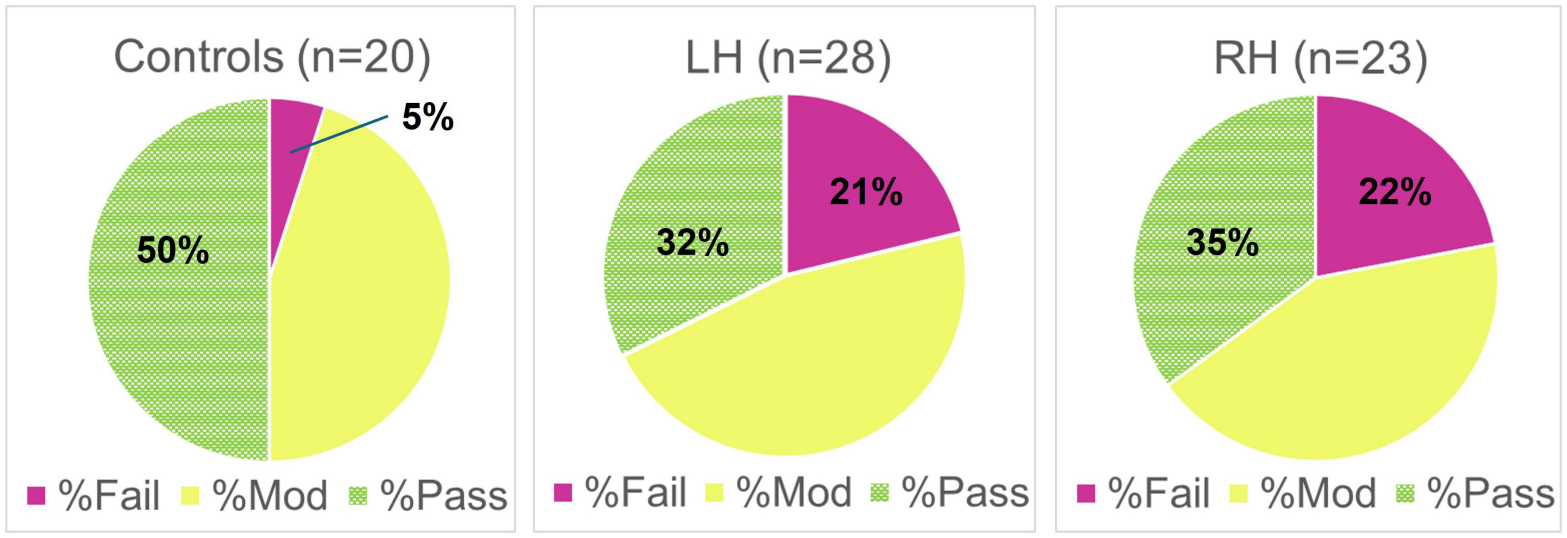
Percentages of failing (Fail), moderately impaired (Mod), and passing (Pass) driver assessment ratings by group (Controls, LH Stroke, RH Stroke).

Stroke survivors were four times as likely to “fail” the driving assessment (LH: 21%, RH: 22%) compared to age-matched controls (5%) (see Figure 2). An ordinal logistic regression analysis of the driver assessment ratings (Fail < Moderate Impairment < Pass) confirmed that the stroke survivors had poorer driver assessment ratings (i.e., more failures) compared to controls [*t*_(68)_ = 1.688, MLE odds ratio of 2.3, odds ratio confidence interval lower bound 1.03, *p* < 0.048]. Spearman rank correlations further revealed that the severity of contralesional attention deficit (CAD) after stroke was positively correlated with poorer ratings on the driving assessment [*rho*_(49)_ = 0.293, *p* = 0.037), indicative of more failures among those with greater contralesional attention deficit. Age, education, visual acuity, simple reaction time, and mean grip strength, however, were not predictive of driver assessment ratings [*F*_(5, 64)_ = 0.548, all *p* > 0.268].

### Driving errors after stroke

**Figure 3:**
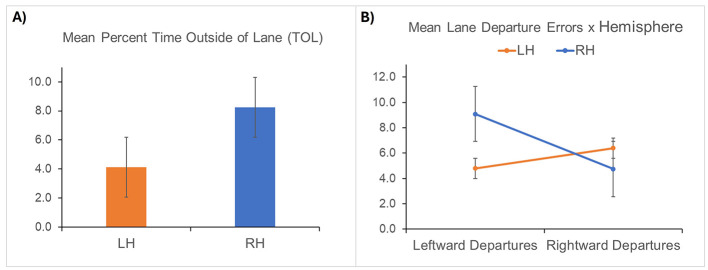
Injured hemisphere had a significant impact on lane positioning errors, shown with standard error bars. **(A)** RH stroke survivors spent a greater proportion of time driving outside of the lane lines (TOL). **(B)** Lesioned hemisphere interacted with direction of lane departures, with lane departures being more prevalent in the contralesional direction, especially among the RH stroke participants who made significantly more leftward (compared to rightward) lane departures.

Analysis of lane positioning errors revealed a significant main effect of hemisphere [*F*_(1, 49)_ = 3.58, *p* = 0.021], with RH stroke survivors exhibiting greater TOL [*F*_(1, 49)_ = 3.90 *p* = 0.054], and making significantly more leftward lane departures [*F*_(1, 49)_ = 7.81, *p* = 0.007]. A repeated-measures ANOVA also revealed a significant interaction between affected hemisphere and direction of lane departures ([*F*_(1, 49)_ = 9.92, *p* = 0.003], see Figure 3 below), revealing more lane departures in the contralesional direction. No other group differences were found for the remaining driving errors (all *p* > 0.220).

### Group characteristics

An ANOVA examining executive functioning errors revealed a significant effect of group [*F*_(2, 68)_ = 7.21, *p* = 0.001], with contrasts revealing greater executive dysfunction among the stroke survivors compared to controls (see Table 2). An ANOVA examining hemisphere, however, revealed that executive functioning errors did not differ significantly between the LH and RH stroke groups [*F*_(1, 49)_ = 2.83, *p* = 0.099]. With respect to presence of aphasia after stroke (based on a WAB score < 93.8), an ANOVA revealed a significantly greater presence of aphasia after a LH stroke [*F*_(1, 49)_ = 7.37, *p* = 0.009] compared to RH stroke. The mean WAB Aphasia Quotient score for the LH group (*M* = 91.88, *SD* = 16.06) was also below the WAB cutoff score of 93.7. The breakdown of aphasia types among the LH stroke survivors was 5 Anomic, 1 Conduction, and 1 Wernicke's aphasia.

**Table 2:** Descriptive data for cognition and driving scores by group, with mean (and range) shown for each measure.

				**AIM score** = **L – R/L** + **R**		
**Group**	**Driving score**	**WAB Aphasia Quotient (AQ)**	**Executive functioning errors**	**Left (L) visual search threshold [ms]**	**Right (R) visual search threshold [ms]**	**Contralesional attention deficit (CAD) severity ratio)**
Controls	9.35 (1–26)	n/a	0.55 (0–5)	376 (61–922)	263 (24–986)	n/a
LH stroke	15.53 (1–139.25)	91.88 (36.5–100)	1.93 (0–8)	383 (59–916)	402 (16–1,428)	1.21 (0.04–4.22)
RH stroke	11.99 (2–34.5)	98.54 (94.5–100)	3.09 (0–8)	568 (74–1,400)	425 (58–800)	1.6 (0.51–4.53)

On average, the LH stroke group had a higher mean driving score (i.e., more driving errors), a mean WAB AQ score below the 93.8 cutoff for performing within normal limits, and slower rightward compared to leftward visual search. In contrast, the RH stroke group made more executive functioning errors, were slowest in leftward visual search, and had a more severe contralesional attention deficit.

**Figure 4:**
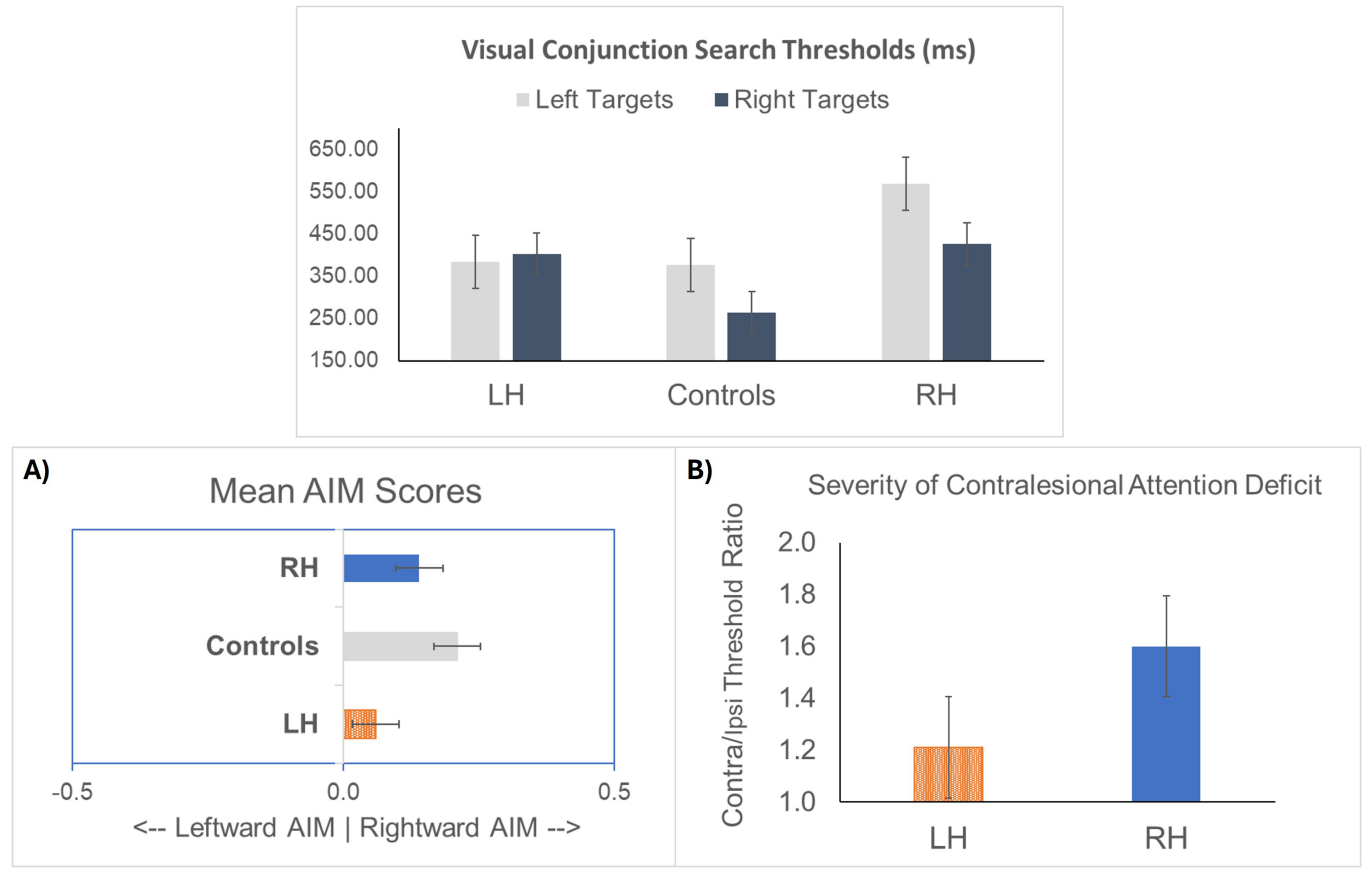
Mean visual search thresholds for left and right targets, shown with standard error bars. **(A)** Attentional Imbalance (AIM) Scores by group. **(B)** Severity of contralesional attention deficit after stroke by affected hemisphere.

ANOVAs examining group differences for left and right visual search thresholds revealed a significant main effect of Group [*F*_(2, 68)_ = 4.625, *p* = 0.013] for left visual search thresholds, with the RH group requiring the longest viewing times for left-sided targets (see Table 2, and left panel, Figure 4). An ANOVA examining the direction of Attentional Imbalance (AIM), however, revealed primarily rightward bias irrespective of Group ([*F*_(2, 68)_ = 1.348, *p* = 0.267], see middle panel Figure 4). With regard to contralesional attention deficits after stroke, LH stroke survivors required 1.2 times longer viewing times on average to detect contralesional (compared to ipsilesional) targets, while RH participants needed 1.6 times longer viewing times (right panel Figure 4). In terms of the frequency of contralesional attention deficits after stroke, 16/23 (70%) of the RH and 13/28 (57%) of the LH stroke participants required more viewing time to accurately detect contralesional targets. The severity of contralesional attention deficit, however, did not differ as a function of hemisphere [*F*_(1, 49)_ = 1.595, *p* = 0.213].

### Correlates of driving performance

**Figure 5:**
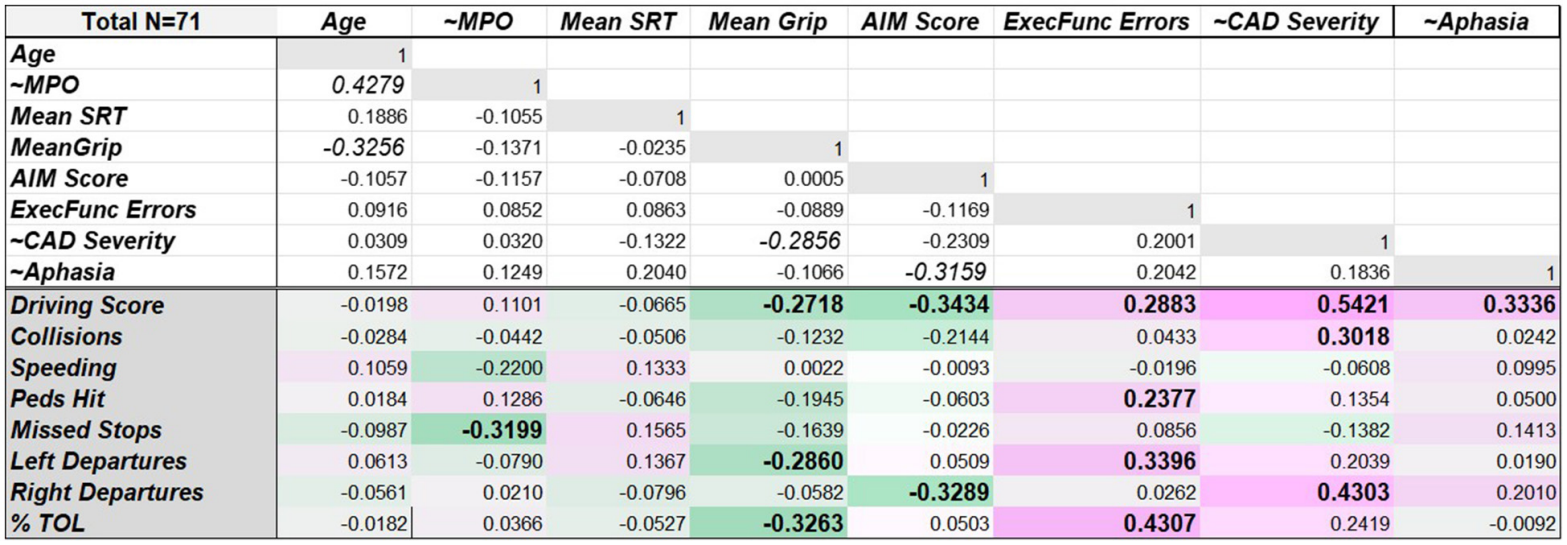
Heat map of the strength of correlation coefficients between demographic, sensorimotor, and cognitive/linguistic factors and driving measures (shown in lower section). Negative correlations with driving measures are shaded in green tones (factors associated with fewer driving errors), while positive correlations are represented in pink tones (factors associated with more driving errors). Bold font indicates statistically significant correlation coefficients. Non-colored correlations above the double solid line were not planned, but show relationships among the various cognitive, sensorimotor and demographic factors. ~Denotes measures only available for stroke participants (*n* = 51).

CAD severity in stroke survivors was positively correlated with overall driving score [*r*_(49)_ = 0.542, *p* < 0.001, 95%CI (0.31, 0.71)], vehicle collisions [*r*_(49)_ = 0.302, *p* = 0.031, 95%CI (0.03, 0.53)], and rightward lane departures [*r*_(49)_ = 0.430, *p* = 0.002, 95%CI (0.18, 0.63)], suggesting that stroke survivors with greater contralesional attentional impairment made more driving errors overall and got into more car accidents (see Figure 5). Within the stroke group, there was also a positive association between presence of aphasia and driving score [*r*_(49)_ = 0.334, *p* = 0.017, 95%CI (0.064, 0.558)]. In addition, executive dysfunction was associated with poorer driving performance across all participants. Specifically, executive functioning errors were positively correlated with driving score [i.e., more driving errors, *r*_(69)_ = 0.288, *p* = 0.015, 95%CI (0.06, 0.49)], numbers of pedestrians hit [*r*_(69)_ = 0.238, *p* = 0.046, 95%CI (0.00, 0.45)], numbers of leftward lane departures [*r*_(69)_ = 0.340, *p* = 0.004, 95%CI (0.12, 0.53)], and TOL [*r*_(69)_ = 0.431, *p* < 0.001, 95%CI (0.22, 0.60)].

In contrast, mean grip strength was negatively correlated with driving errors. Specifically, greater mean grip strength (Figure 5) was associated with lower (better) driving scores [*r*_(69)_ = −0.272, *p* = 0.022, 95% CI (−0.47, −0.04)], fewer leftward lane departures [*r*_(69)_ = −0.286, *p* = 0.016, 95%CI (−0.49, −0.06)], and less TOL [*r*_(69)_ = −0.326, *p* = 0.005, 95%CI (−0.52, −0.10)]. In addition, AIM score was negatively correlated with driving scores [*r*_(69)_ = −0.343, *p* = 0.003, 95% CI (−0.53, −0.12)] and rightward lane departures [*r*_(69)_ = −0.329, *p* = 0.005, 95% CI (−0.52, −0.10)], suggesting that individuals with greater attention toward the right side achieved better driving scores and made fewer rightward lane departures. Finally, a significant negative correlation was observed between months post-stroke and missed stops [*r*_(49)_ = −0.320, *p* = 0.022, 95% CI (−0.55, −0.05)], suggesting fewer missed stops with increasing time post-stroke. Potential multicollinearity among the various demographic, cognitive, and sensorimotor measures can be seen in the correlations shown in the top portion of Figure 5. As might be expected, mean grip strength correlated negatively with age [*r*_(69)_ = −0.326, *p* = 0.006, 95% CI (−0.519, −0.010)]. Mean grip strength also correlated negatively with CAD severity [*r*_(49)_ = −0.286, *p* = 0.042, 95% CI (−0.520, −0.011)], suggesting that reduced grip strength in either hand may be associated with larger contralesional attention deficits. Finally, presence of aphasia was negatively correlated with AIM Score [*r*_(49)_ = −0.316, *p* = 0.024, 95% CI (−0.544, −0.044)], indicating a relationship between presence of aphasia and reduced rightward attention. Importantly, there were no significant correlations between AIM score, CAD severity, and executive functioning performance.

### Factors predictive of driving performance

In order to examine which factors were most predictive of driving performance, a multi-step linear regression analysis was conducted. In the first step regression model, we analyzed the winsorized driving scores from the entire sample including factors of group (LH, RH, Control), attentional imbalance (AIM Score), and executive functioning errors (AIC = 491.5). This analysis revealed that AIM Score was a significant predictor of driving scores [*B* = −6.268, *SE* = 2.887, CI = (−11.926, −0.611), *p* = 0.034] with a medium effect size (η^2^ = 0.08). Specifically, greater attention to the right was predictive of better driving scores. In addition, there was a modest positive association between executive functioning errors and driving score [*B* = 0.673, *SE* = 0.409, CI = (−0.130, 1.475), *p* = 0.105)], reflecting a small effect size (η^2^ = 0.04), but suggesting that greater executive dysfunction may be associated with more driving errors. Group, on the other hand, was not predictive of driving score (*p* > 0.6). Thus, the group factor was dropped in the second step of the model while sensorimotor factors (simple reaction time and mean grip strength) were added. This model (AIC = 481.39) again revealed that AIM Score [*B* = −6.511, *SE* = 2.789, CI (−11.978, −1.045), *p* = 0.023] was a significant predictor of driving scores, with medium effect size (η^2^ = 0.09). In addition, a significant negative relationship between mean grip strength and driving score was identified [*B* = −0.179, *SE* = 0.086, CI = (−0.349, −0.010), *p* = 0.042, η^2^ = 0.06] in that greater grip strength predicted better driving scores. Simple reaction time was not predictive of driving score (*p* = 0.795). A small effect of executive dysfunction was again noted as a trend positive relationship between driving score and executive functioning errors [*B* = 0.685, *SE* = 0.385, CI = (−0.070, 1.441) *p* = 0.080, η^2^ = 0.05].

A final linear regression analysis, restricted to the stroke survivors' driving scores, was also conducted. This analysis included all factors that achieved a criterion value of *p* < 0.30 in the prior analysis (AIM score, executive functioning errors, and mean grip strength) along with three additional stroke-specific variables (i.e., CAD severity, presence of aphasia, and months post-stroke onset). This model (AIC = 357.69) revealed that both the severity of contralesional attention deficit [CAD: *B* = 2.814, *SE* = 1.040, *CI* = (0.777, 4.852), *p* = 0.010, η^2^ = 0.15] and the direction of attentional imbalance [AIM score: *B* = −7.718, *SE* = 3.603, *CI* = (−14.780, −0.655), *p* = 0.038, η^2^ = 0.18] were predictive of post-stroke driving scores, with large effect size. In contrast, presence of aphasia and MPO were not reliably predictive of driving score (*p* = 0.688 and *p* = 0.266, respectively).

### Simulator sickness

Neither baseline scores on the Simulator Sickness Questionnaire [*SE* = 0.008, *CI* = (−0.03, 0.01), *p* = 0.129], nor those obtained from questionnaires administered just before [*SE* = 0.008, *CI* = (−0.02, 0.01), *p* = 0.402] or after the driving assessment [*SE* = 0.005, *CI* = (−0.01, 0.01), *p* = 0.377] were predictive of driving score.

## Discussion

This study evaluated the degree to which visuospatial attention deficits, executive dysfunction, and presence of aphasia after stroke were predictive of impaired driving and associated with different types of driving errors in LH vs. RH stroke survivors. With regard to hemisphere, RH stroke survivors exhibited significantly more leftward lane departure errors and spent proportionally more time driving outside of the lane lines, indicating greater difficulty maintaining and correcting lane position. LH stroke survivors, on the other hand, tended to make more errors on the right side of the lane (i.e., rightward lane departures) and were equally as likely to fail a simulated driving assessment compared to RH stroke survivors. Importantly, comparable levels of executive dysfunction and contralesional attention deficits were observed in the LH and RH stroke groups.

In terms of cognitive predictors of driving performance in stroke survivors, regression analyses revealed that the direction and severity of attentional imbalances were the strongest predictors of post-stroke driving performance. The direction of attentional imbalance (measured via AIM score) indicated that greater rightward attention predicted better driving scores, with a large effect size (Cohen, 1988). In addition, the severity of contralesional attentional deficit (CAD) also showed a large effect size, with more severe contralesional attention deficits resulting in poorer driving scores, including increased collisions and rightward lane departures.

In sum, we found that stroke survivors with a more pronounced contralesional attention deficit performed more poorly on the simulated driving assessment, including higher failure rates, poorer overall driving scores, increased collisions, and a greater tendency to make rightward lane departures. An interaction between hemisphere of injury and direction of lane deviations further confirmed that stroke survivors had a greater tendency to veer out of the lane in the contralesional direction. These findings emphasize the critical role of attentional impairments, particularly among stroke survivors, who may have mild to moderate lateralized deficits that can persist for many years after unilateral brain injury. Importantly, the method for quantifying severity of contralesional attention deficits in this study was based on each individual's threshold for detecting a left vs. right-sided target and was thus not confounded with motoric deficits. Such quantification of contralesional attention deficits after stroke is not currently included in driving assessments but would clearly help to identify individuals who may be at greater risk to themselves and others while driving.

Although not predictive of overall driving scores, executive dysfunction was found to correlate significantly with specific types of driving errors, including collisions with pedestrians and leftward lane departures. Notably, executive functioning errors also correlated significantly with TOL, or the proportion of time spent driving outside of the lane lines. This suggests that executive dysfunction may disrupt the ability to recognize and correct mistakes, such as poor lane position. In the sample studied here, stroke survivors (irrespective of hemisphere of stroke) made significantly more executive functioning errors than age-matched controls and were four times as likely to fail the simulated driving assessment. These findings corroborate other research underscoring the importance of executive functioning in driving safety (Motta et al., 2014) as well as reports that executive function and visuospatial cognition are often more predictive of driving errors than basic vision in older adults (Aksan et al., 2013). The current findings further emphasize the importance of evaluating executive functioning in assessments of fitness to drive after acquired brain injury.

Of note, rightward AIM (attentional bias favoring the right side of the visual environment) was associated with fewer driving errors across all participants, including healthy age-matched controls. Notably, the measure of rightward AIM was obtained from a task that did not involve any driving. Nonetheless, greater rightward AIM was associated with better driving performance (i.e., better driving scores, fewer rightward lane departures), suggesting that rightward attention may be a protective factor for driving. This extends findings from prior research conducted in healthy older drivers that has found visual attention to be a better predictor of on-road driving performance than age (Baldock et al., 2007). Rightward attention while driving is likely beneficial because all speed limit signs and stop signs are presented on the right side in the driving simulator, consistent with their placement on US roadways. Not surprisingly, the LH stroke group (which was most deficient in rightward attention and had the lowest AIM score) had the highest mean driving score (i.e. most driving errors).

Unlike many stroke studies that exclude people with aphasia (Shiggins et al., 2024), the current study included individuals with aphasia in order to evaluate the potential impact of language impairment on driving. Including individuals with aphasia has important ramifications because social isolation often accompanies impairments in speech and language, and driving offers many people a principal means of engaging in activities and social outings (Griffen et al., 2009). Interestingly, presence of aphasia was correlated with poorer driving scores and reduced rightward attention (AIM score). Regression analyses, however, revealed that visuospatial attention factors were the better predictors of driving scores. More specifically, the direction and severity of attention imbalances after stroke were significant predictors of driving scores, while presence of aphasia (as determined by the WAB-R) was not. While it has been reported that language ability can impact road sign recognition (Donovan et al., 2015), it is also possible that reduced rightward attention in participants with aphasia may contribute to driving errors. Notably, stop signs and posted speed limits were always located on the right side of the roadway in the simulator (as they are on US roadways). Nevertheless, presence of aphasia as determined by the WAB cut-off score conveys limited sensitivity, so future studies using more sensitive measures of aphasia, including latent aphasia (Salis et al., 2021; Fromm et al., 2017) may reveal a greater impact of language ability on driving.

The findings from this study thus underscore how driving performance can be negatively impacted by both the direction and severity of visuospatial attention deficits resulting from a stroke involving either cerebral hemisphere. Although earlier work by Anderson and colleagues (Anderson et al., 2012) suggested that vision and cognitive function are distinct in their impact on driving in older adults with and without neurological disease, no studies have attempted to disentangle deficits in visuospatial *attention* from impairments in sensorimotor or executive functioning. The fact that both RH and LH stroke can result in contralesional visuospatial attention deficits that persist even years post-stroke suggests a critical need for more reliable and standardized methods of quantifying lateralized attention deficits prior to getting back behind the wheel. In addition, the current findings also underscore the importance of assessing executive functioning in post-stroke driving and suggest that factors such as age, simple reaction time, and aphasia may play a lesser role.

## Limitations

In the current study, driving performance was assessed in a simulator rather than an on-road driving assessment. Although on-road driving evaluations are typically preferred as the final measure of driving fitness (D'apolito et al., 2013), driving simulators convey several advantages. First, simulated driving can better emulate complex and challenging driving situations that often do not arise during an on-road evaluation, such as pedestrians unexpectedly entering the roadway, multiple changes in posted speed limits, construction zones, and vehicles that suddenly merge into or block a portion of the roadway. Second, driving simulation offers a standardized, consistent, and unbiased method for evaluating driving performance. On a practical level, simulated driving assessments are relatively low-cost and safer than on-road driving assessments (Rosen and Wachtel, 2004), particularly when assessing individuals who may be compromised due to brain injury or health limitations. Importantly, simulated driving has also been reported to be highly accurate (91%) in predicting on-road driving performance (Akinwuntan et al., 2011), and driving errors between the two platforms have been reported to align moderately well, especially for lane positioning errors (Aksan et al., 2016).

Another limitation of the current study is that we did not include any individuals in the acute phase of stroke (Hird et al., 2015). Instead, we focused on individuals who ranged from at least 3 months to several years post-stroke so that symptoms were stable and participants would be able to manage the driving simulator. Additional studies focusing specifically on the first 6 months post-stroke may reveal more pronounced differences and provide additional insights into the importance of time post-stroke when considering a return to driving. A related limitation is that the current study sample included few individuals with moderate to severe aphasia and was limited to fluent type aphasias (e.g., no Broca's aphasia). Thus, due to the statistical constraints of the dataset, we used a binarized variable (presence of aphasia, based on the standardized WAB-R cutoff). Future work is planned with a larger sample of individuals with aphasia so that we can analyze the impact of the whole range of aphasia spectrum based on continuous aphasia severity scores (WAB AQ) and aphasia subtype.

Last, the current study participants were all U.S. Veterans, who are at particular risk of social isolation/loneliness, which may be exacerbated by the loss of functional independence that is associated with an inability to drive. While Veterans can also be more likely to have comorbid health conditions than the general population, participants in the current study were carefully screened to meet strict inclusion/exclusion criteria which enhances the generalizability of the findings. However, the Veteran stroke survivor population is predominantly male, which limited our recruitment of female participants. Among older drivers, there are discrepant reports regarding whether males or females are responsible for more motor vehicle fatalities (Insurance Institute for Highway Safety Highway Loss Data Institute, 2025; National Center for Statistics Analysis, 2024), but some evidence suggests that older females are less likely to drive over the speed limit (Bauer et al., 2003). Importantly, all participants in our study were forewarned that speed limits would be strictly enforced in the simulator.

## Conclusions

In summary, this study assessed driving performance in right vs. left hemisphere stroke patients on a driving simulator which offers a safe, challenging, and cost-effective means of assessing driving skills. The results from the study have ramifications for clinicians, stroke survivors, Veterans with acquired brain injury, and individuals with aphasia. In particular, the findings shed light on the unique challenges faced by stroke survivors while driving and serve to inform both clinicians and their patients of potential risks and vulnerabilities that can occur after unilateral brain injury, as well as factors that may be of lesser consequence. Notably, both the direction and severity of visuospatial attention deficits were found to be strong predictors of post-stroke driving performance in the population studied here. Although not as predictive, executive dysfunction was also found to correlate with specific types of driving errors including collisions with pedestrians, lane departures, and time spent outside of the lane lines.

Given the large effect sizes of the direction and severity of contralesional attentional imbalances on post-stroke driving, these results further suggest that driving rehabilitation efforts that target specific cognitive skills such as increased visuospatial attention and greater vigilance of visual information on the right side of the roadway may prove highly beneficial to enhancing driving safety and functional independence among both older drivers and stroke survivors. Among stroke survivors in particular, reducing the amount of distracting information that is present or directing attention to either the left or right side of the driving environment could be particularly helpful depending on the direction and severity of their visuospatial attention deficits. In addition, rehabilitation efforts that focus on driving skills that are shown to correlate with specific cognitive impairments (e.g. staying in your lane, monitoring speed, and avoiding roadway obstacles) may also be especially beneficial. Current work at our center includes a clinical trial investigating the effectiveness of driving simulator training that focusses on lane positioning, speed management, collision avoidance, and divided attention while driving.

## Data Availability

The datasets presented in this article are not readily available because sharing/disclosure of this Veteran dataset outside of VA is not approved by our local IRB. Requests to access the datasets should be directed to Krista.Parker@va.gov.

## References

[B1] Akinwuntan A. E. De Weerdt W. Feys H. Pauwels J. Baten G. Arno P. . (2005). Effect of simulator training on driving after stroke: a randomized controlled trial. Neurology 65, 843–850. doi: 10.1212/01.wnl.0000171749.71919.fa 16186521

[B2] Akinwuntan A. E. Devos H. Verheyden G. Baten G. Kiekens C. Feys H. . (2010). Retraining moderately impaired stroke survivors in driving-related visual attention skills. Top. Stroke Rehabil. 17, 328–336. doi: 10.1310/tsr1705-328 21131257

[B3] Akinwuntan A. E. Gantt D. Gibson G. Kimmons K. Ross V. Rosen P. . (2011). “The United States version of the Stroke Drivers' Screening Assessment battery: a report of preliminary findings,” in Proceedings of the Sixth International Driving Symposium on Human Factors in Driver Assessment, Training and Vehicle Design (Lake Tahoe, CA).

[B4] Akinwuntan A. E. Wachtel J. Rosen P. (2012). Driving simulation for evaluation and rehabilitation of driving after stroke. J. Stroke Cerebrovasc. Dis. 21, 478–486. doi: 10.1016/j.jstrokecerebrovasdis.2010.12.001 21236698

[B5] Aksan N. Dawson J. D. Emerson J. L. Yu L. Uc E. Y. Anderson S. W. . (2013). Naturalistic distraction and driving safety in older drivers. Hum. Factors 55, 841–853. doi: 10.1177/0018720812465769 23964422 PMC3880225

[B6] Aksan N. Hacker S. D. Sager L. Dawson J. Anderson S. Rizzo M. (2016). Correspondence between simulator and on-road drive performance: implications for assessment of driving safety. Geriatrics 1:8. doi: 10.3390/geriatrics1010008 28649572 PMC5479070

[B7] Anderson S. W. Aksan N. Dawson J. D. Uc E. Y. Johnson A. M. Rizzo M. (2012). Neuropsychological assessment of driving safety risk in older adults with and without neurologic disease. J. Clin. Exp. Neuropsychol. 34, 895–905. doi: 10.1080/13803395.2011.630654 22943767 PMC3910382

[B8] Aufman E. L. Bland M. D. Barco P. P. Carr D. B. Lang C. E. (2013). Predictors of return to driving after stroke. Am. J. Phys. Med. Rehabil. 92, 627–634. doi: 10.1097/PHM.0b013e318282bc0d 23370577 PMC3689872

[B9] Azouvi P. Samuel C. Louis-Dreyfus A. Bernati T. Bartolomeo P. Beis J. M. . (2002). Sensitivity of clinical and behavioural tests of spatial neglect after right hemisphere stroke. J. Neurol. Neurosurg. Psychiatry 73, 160–166. doi: 10.1136/jnnp.73.2.160 12122175 PMC1737990

[B10] Bach M. (1996). The “Freiburg Visual Acuity Test” – automatic measurement of visual acuity. Optom. Vis. Sci. 73, 49–53. doi: 10.1097/00006324-199601000-00008 8867682

[B11] Bach M. (2007). The Freiburg Visual Acuity Test – variability unchanged by post-hoc re-analysis. Graefes Arch. Clin. Exp. Ophthalmol. 245, 965–971. doi: 10.1007/s00417-006-0474-4 17219125

[B12] Baldock M. R. J. Mathias J. McLean J. Berndt A. (2007). Visual attention as a predictor of on-road driving performance of older drivers. Aust. J. Psychol. 59, 159–168. doi: 10.1080/00049530701458035

[B13] Barkley R. A. Murphy K. R. Dupaul G. I. Bush T. (2002). Driving in young adults with attention deficit hyperactivity disorder: knowledge, performance, adverse outcomes, and the role of executive functioning. J. Int. Neuropsychol. Soc. 8, 655–672. doi: 10.1017/S1355617702801345 12164675

[B14] Bauer M. J. Adler G. Kuskowski M. A. Rottunda S. (2003). The influence of age and gender on the driving patterns of older adults. J. Women Aging 15, 3–16. doi: 10.1300/J074v15n04_02 14750586

[B15] Behrmann M. Tipper S. P. (1999). Attention accesses multiple reference frames: evidence from visual neglect. J. Exp. Psychol. Hum. Percept. Perform. 25, 83–101. doi: 10.1037/0096-1523.25.1.83 10069027

[B16] Beis J. M. Keller C. Morin N. Bartolomeo P. Bernati T. Chokron S. . (2004). Right spatial neglect after left hemisphere stroke qualitative and quantitative study. Neurology 63, 1600–1605. doi: 10.1212/01.WNL.0000142967.60579.32 15534242

[B17] Berger P. Mensh S. (2011). How to succeed in patient-centered aphasia therapy and measure results. Top. Stroke Rehabil. 18, 285–291. doi: 10.1310/tsr1803-285 21642066

[B18] Blini E. Romeo Z. Spironelli C. Pitteri M. Meneghello F. Bonato M. . (2016). Multi-tasking uncovers right spatial neglect and extinction in chronic left-hemisphere stroke patients. Neuropsychologia 92, 147–157. doi: 10.1016/j.neuropsychologia.2016.02.028 26948071

[B19] Bonato M. (2012). Neglect and extinction depend greatly on task demands: a review. Front. Hum. Neurosci. 6:195. doi: 10.3389/fnhum.2012.00195 22822394 PMC3398353

[B20] Breen J. Andrusin J. Ferlito T. (2016). Characteristics of stroke survivors treated in an interdisciplinary outpatient rehabilitation program who return to work. Stroke 47:WP152. doi: 10.1161/str.47.suppl_1.wp152

[B21] Breen J. Andrusin J. Ferlito T. Hobbs S. (2017). Characteristics and estimated rehabilitation costs for stroke survivors treated in a community-based interdisciplinary outpatient rehabilitation program who return to work. Stroke 48:TP148. doi: 10.1161/str.48.suppl_1.tp148

[B22] Breen J. C. (2021). Community-based outpatient stroke rehabilitation program achieves excellent return to work outcomes: characteristics and rehabilitation outcomes of stroke survivors who return to work. Stroke 52:P209. doi: 10.1161/str.52.suppl_1.P209

[B23] Cohen J. (1988). Statistical Power Analysis for the Behavioral Sciences. Hillsdale, NJ: Lawrence Erlbaum Associates.

[B24] D'apolito A. C. Massonneau A. Paillat C. Azouvi P. (2013). Impact of brain injury on driving skills. Ann. Phys. Rehabil. Med. 56, 63–80. doi: 10.1016/j.rehab.2012.12.002 23375369

[B25] Daugherty J. Sarmiento K. Breiding M. (2023). Comparison of self-reported lifetime concussions and mild traumatic brain injuries among adults. Brain Inj. 37, 222–229. doi: 10.1080/02699052.2023.2175909 36760062 PMC10409868

[B26] Demireva P. McInerney K. Suhr J. (2012). The importance of executive functioning ability in simulated driving performance among healthy, community-dwelling, older adults. Arch. Clin. Neuropsychol. 27:673.

[B27] Deouell L. Y. Sacher Y. Soroker N. (2005). Assessment of spatial attention after brain damage with a dynamic reaction time test. J. Int. Neuropsychol. Soc. 11, 697–707. doi: 10.1017/S1355617705050824 16248905

[B28] Devos H. Akinwuntan A. E. Nieuwboer A. Ringoot I. Van Berghen K. Tant M. . (2010). Effect of simulator training on driving after stroke: a 5-year follow-up of a randomized trial. Neurorehabil. Neural Repair 24, 843–850. doi: 10.1177/1545968310368687 20656965

[B29] Devos H. Akinwuntan A. E. Nieuwboer A. Truijen S. Tant M. De Weerdt W. (2011). Screening for fitness to drive after stroke: a systematic review and meta-analysis. Neurology 76, 747–756. doi: 10.1212/WNL.0b013e31820d6300 21339502

[B30] Devos H. Morgan J. C. Onyeamaechi A. Bogle C. A. Holton K. Kruse J. . (2016). Use of a driving simulator to improve on-road driving performance and cognition in persons with Parkinson's disease: a pilot study. Aust. Occup. Ther. J. 63, 408–414. doi: 10.1111/1440-1630.12263 27071987

[B31] Devos H. Verheyden G. Van Gils A. Tant M. Akinwuntan A. E. (2015). Association between site of lesion and driving performance after ischemic stroke. Top. Stroke Rehabil. 22, 246–252. doi: 10.1179/1074935714Z.0000000018 26258450

[B32] Donovan N. J. Savage M. C. Varnado C. Brown C. (2015). Road sign Recognition During Computer Testing Versus Driving Simulator Performance for Stroke and Stroke + *Aphasia Groups*. Fayetteville, AR: Maritime Transportation Research and Education Centre.

[B33] FindLaw (2023). California Code Health and Safety Code – HSC § 103900. Available online at: https://codes.findlaw.com/ca/health-and-safety-code/hsc-sect-103900/ (Accessed January 1, 2023).

[B34] Fisk G. D. Owsley C. Pulley L. V. (1997). Driving after stroke: driving exposure, advice, and evaluations. Arch. Phys. Med. Rehabil. 78, 1338–1345. doi: 10.1016/S0003-9993(97)90307-5 9421988

[B35] Fromm D. Forbes M. Holland A. Dalton S. G. Richardson J. MacWhinney B. (2017). Discourse characteristics in aphasia beyond the Western Aphasia Battery cutoff. Am. J. Speech Lang. Pathol. 26, 762–768. doi: 10.1044/2016_AJSLP-16-0071 28505222 PMC5829792

[B36] Gasne C. Ranchet M. Evennou M. Delebecque M. C. Hoang I. Coignard P. . (2024). Fitness-to-drive recommendations in post-stroke patients: a retrospective study. J. Stroke Cerebrovasc. Dis. 33:107781. doi: 10.1016/j.jstrokecerebrovasdis.2024.107781 38772498

[B37] Gillen G. Rubio K. B. (2011). “Treatment of cognitive perceptual deficits: a function-based approach,” in Stroke Rehabilitation: A Function-Based Approach, ed. G. Gillen (New York, NY: Elsevier Mosby).

[B38] Griffen J. A. Rapport L. J. Coleman Bryer R. Scott C. A. (2009). Driving status and community integration after stroke. Top. Stroke Rehabil. 16, 212–221. doi: 10.1310/tsr1603-212 19632966

[B39] Hartje W. Willmes K. Pach R. Hannen P. Weber E. (1991). Driving ability of aphasic and non-aphasic brain-damaged patients. Neuropsychol. Rehabil. 1, 161–174. doi: 10.1080/09602019108520162

[B40] Heilman K. M. Valenstein E. Watson R. T. (2000). Neglect and related disorders. Semin. Neurol. 20, 463–470. doi: 10.1055/s-2000-13179 11149702

[B41] Helm-Estabrooks N. (2018). “Cognitive linguistic quick test,” in Encyclopedia of Clinical Neuropsychology (Cham: Springer), 854–857.

[B42] Hird M. A. Vesely K. A. Christie L. E. Alves M. A. Pongmoragot J. Saposnik G. . (2015). Is it safe to drive after acute mild stroke? A preliminary report. J. Neurol. Sci. 354, 46–50. doi: 10.1016/j.jns.2015.04.043 26004673

[B43] Insurance Institute for Highway Safety and Highway Loss Data Institute (2025). Fatality Facts 2023 Report. Available online at: https://www.iihs.org/research-areas/fatality-statistics/detail/older-people (Accessed February 2, 2026).

[B44] Kennedy R. S. Lane N. E. Berbaum K. S. Lilienthal M. G. (1993). Simulator sickness questionnaire: an enhanced method for quantifying simulator sickness. Int. J. Aviat. Psychol. 3, 203–220. doi: 10.1207/s15327108ijap0303_3

[B45] Kertesz A. (2007). Western Aphasia Battery–Revised. San Antonio, TX: PsychCorp.

[B46] Lee N. Tracy J. Bohannon R. W. Ahlquist M. (2003). Driving resumption and its predictors after stroke. Conn. Med. 67, 387–392. 14502860

[B47] Legh-Smith J. Wade D. T. Hewer R. L. (1986). Driving after a stroke. J. R. Soc. Med. 79, 200–203. doi: 10.1177/014107688607900404 3701764 PMC1290273

[B48] List A. Brooks J. L. Esterman M. Flevaris A. Landau A. N. Bowman G. . (2008). Visual hemi-spatial neglect, re-assessed. J. Int. Neuropsychol. Soc. 14, 243–256. doi: 10.1017/S1355617708080284 18282322 PMC2573467

[B49] Lundqvist A. Gerdle B. Ronnberg J. (2000). Neuropsychological aspects of driving after a stroke—in the simulator and on the road. Appl. Cogn. Psychol. 14, 135–150. doi: 10.1002/(SICI)1099-0720(200003/04)14:2<135::AID-ACP628>3.0.CO;2-SDigital

[B50] Marshall S. C. Molnar F. Man-Son-Hing M. Blair R. Brosseau L. Finestone H. M. . (2007). Predictors of driving ability following stroke: a systematic review. Top. Stroke Rehabil. 14, 98–114. doi: 10.1310/tsr1401-98 17311796

[B51] McGwin G. Jr. Sims R. V. Pulley L. Roseman J. M. (2000). Relations among chronic medical conditions, medications, and automobile crashes in the elderly: a population-based case-control study. Am. J. Epidemiol. 152, 424–431. doi: 10.1093/aje/152.5.424 10981455

[B52] Mesulam M. (2002). Functional Neuroanatomy of Attention and Neglect: From Neurons to Networks. New York, NY: Oxford University Press.

[B53] Motta K. Lee H. Falkmer T. (2014). Post-stroke driving: examining the effect of executive dysfunction. J. Saf. Res. 49, 33–e1. doi: 10.1016/j.jsr.2014.02.005 24913483

[B54] National Center for Statistics and Analysis (2024). Older population: 2022 data (Traffic Safety Facts. Report No. DOT HS 813 616). Washington, DC: National Highway Traffic Safety Administration.

[B55] Nouri F. M. Lincoln N. B. (1992). Validation of a cognitive assessment: predicting driving performance after stroke. Clin. Rehabil. 6, 275–281. doi: 10.1177/026921559200600402

[B56] Nouri F. M. Tinson D. J. Lincoln N. B. (1987). Cognitive ability and driving after stroke. Int. Disabil. Stud. 9, 110–115. doi: 10.3109/03790798709166334 3429395

[B57] Oren N. Soroker N. Deouell L. Y. (2013). Immediate effects of exposure to positive and negative emotional stimuli on visual search characteristics in patients with unilateral neglect. Neuropsychologia 51, 2729–2739. doi: 10.1016/j.neuropsychologia.2013.09.033 24080263

[B58] Park M. O. (2015). A comparison of driving errors in patients with left or right hemispheric lesions after stroke. J. Phys. Ther. Sci. 27, 3469–3471. doi: 10.1589/jpts.27.3469 26696720 PMC4681927

[B59] Perrier M. J. Korner-Bitensky N. Petzold A. Mayo N. (2010). The risk of motor vehicle crashes and traffic citations post stroke: a structured review. Top. Stroke Rehabil. 17, 191–196. doi: 10.1310/tsr1703-191 20797963

[B60] Petzold A. Korner-Bitensky N. Rochette A. Teasell R. Dawson A. Binns M. (2010). Driving poststroke: problem identification, assessment use, and interventions offered by Canadian occupational therapists. Top. Stroke Rehabil. 17, 371–379. doi: 10.1310/tsr1705-371 21131262

[B61] Posner M. I. Walker J. A. Friedrich F. J. Rafal R. D. (1984). Effects of parietal injury on covert orienting of attention. J. Neurosci. 4, 1863–1874. doi: 10.1523/JNEUROSCI.04-07-01863.1984 6737043 PMC6564871

[B62] Reston J. Turkelson C. Coleman P. Belinson S. (2008). Stroke and Commercial Motor Vehicle Driver Safety Executive Summary. Evidence Report presented to The Federal Motor Carrier Safety Administration. Butler Pike, PA: ECRI Institute.

[B63] Ringman J. M. Saver J. L. Woolson R. F. Clarke W. R. Adams H. P. (2004). Frequency, risk factors, anatomy, and course of unilateral neglect in acute stroke cohort. Neurology 63, 468–474. doi: 10.1212/01.WNL.0000133011.10689.CE 15304577

[B64] Rosen P. N. Wachtel J. (2004). Driving simulation in the clinical setting: utility for testing and treatment. Adv. Transp. Stud. 2004, 91–96. Available online at: https://www.atsinternationaljournal.com/2004-issues/driving-simulation-in-the-clinical-setting-utility-for-testing-and-treatment/ (Accessed July 10, 2025).

[B65] Roy E. A. Reuter-Lorenz P. Roy L. G. Copland S. Moscovitch M. (1987). Unilateral attention deficits and hemispheric asymmetries in the control of attention. Adv. Psychol. 45, 25–39. doi: 10.1016/S0166-4115(08)61707-0

[B66] Salis C. Martin N. Reinert L. (2021). Sentence recall in latent and anomic aphasia: an exploratory study of semantics and syntax. Brain Sci. 11:230. doi: 10.3390/brainsci11020230 33673290 PMC7917924

[B67] Schanke A. K. Sundet K. (2000). Comprehensive driving assessment: neuropsychological testing and on-road evaluation of brain injured patients. Scand. J. Psychol. 41, 113–121. doi: 10.1111/1467-9450.00179 10870430

[B68] Schendel K. Dronkers N. F. Turken U. (2016). Not just language: persisting lateralized visuospatial impairment after left hemisphere stroke. J. Int. Neuropsychol. Soc. 22, 695–704. doi: 10.1017/S1355617716000515 27329557 PMC10773962

[B69] Schendel K. L. Robertson L. C. (2002). Using reaction time to assess patients with unilateral neglect and extinction. J. Clin. Exp. Neuropsychol. 27, 941–950. doi: 10.1076/jcen.24.7.941.8390 12647770

[B70] Shewan C. M. Kertesz A. (1980). Reliability and validity characteristics of the Western Aphasia Battery (WAB). J. Speech Hear. Disord. 45, 308–324. doi: 10.1044/jshd.4503.308 7412225

[B71] Shiggins C. Ryan B. Dewan F. Bernhardt J. O'Halloran R. Power E. . (2024). Inclusion of people with aphasia in stroke trials: a systematic search and review. Arch. Phys. Med. Rehabil. 105, 580–592. doi: 10.1016/j.apmr.2023.06.010 37394026

[B72] Shimonaga K. Hama S. Tsuji T. Yoshimura K. Nishino S. Yanagawa A. . (2021). The right hemisphere is important for driving-related cognitive function after stroke. Neurosurg. Rev. 44, 977–985. doi: 10.1007/s10143-020-01272-9 32162124

[B73] Silverberg N. D. Iverson G. L. Cogan A. Dams-O-Connor K. Delmonico R. Graf M. J. P. . (2023). The American Congress of Rehabilitation Medicine diagnostic criteria for mild traumatic brain injury. Arch. Phys. Med. Rehabil. 104, 1343–1355. doi: 10.1016/j.apmr.2023.03.036 37211140

[B74] Sims J. Rouse-Watson S. Schattner P. Beveridge A. Jones K. M. (2012). To drive or not to drive: assessment dilemmas for GPs. Int. J. Fam. Med. 2012:417512. doi: 10.1155/2012/417512 22295200 PMC3265213

[B75] Sotokawa T. Nasu S. Ikuta J. Sonohara K. (2024). Evaluation of driving fitness using driving simulators in patients with right-hemisphere damage: an unmatched case-control study. Top. Stroke Rehabil. 31, 167–177. doi: 10.1080/10749357.2023.2235797 37454345

[B76] Spreij L. A. Ten Brink A. F. Visser-Meily J. M. A. Nijboer T. C. W. (2020). Simulated driving: the added value of dynamic testing in the assessment of visuospatial neglect after stroke. J. Neuropsychol. 14, 28–45. doi: 10.1111/jnp.12172 30325578 PMC7154737

[B77] Stone S. P. Wilson B. Wroot A. Halligan P. W. Lange L. S. Marshall J. C. . (1991). The assessment of visuo-spatial neglect after acute stroke. J. Neurol. Neurosurg. Psychiatry 54, 345–350. doi: 10.1136/jnnp.54.4.345 2056321 PMC488491

[B78] Tsao C. W. Aday A. W. Almarzooq Z. I. Anderson C. A. Arora P. Avery C. L. . (2023). Heart disease and stroke statistics-−2023 update: a report from the American Heart Association. Circulation 147, e93–e621. doi: 10.1161/CIR.0000000000001123 36695182 PMC12135016

[B79] U.S. Centers for Disease Control and Prevention (2023). Stroke Facts. Available online at: https://www.cdc.gov/stroke/data-research/facts-stats/ (Accessed August 20, 2023).

[B80] Wallace H. E. Gullo H. L. Copland D. A. Rotherham A. Wallace S. J. (2024). Does aphasia impact on return to driving after stroke? A scoping review. Disabil. Rehabil. 46, 5977–6000. doi: 10.1080/09638288.2024.2317989 38415619

[B81] Woods D. L. Pebler P. Johnson D. Herron T. Hall K. Blank M. . (2024). The California Cognitive Assessment Battery (CCAB). Front. Hum. Neurosci. 17:1305529. doi: 10.3389/fnhum.2023.1305529 38273881 PMC10809797

[B82] Yu S. Muhunthan J. Lindley R. Glozier N. Jan S. Anderson C. . (2016). Driving in stroke survivors aged 18-65 years: the Psychosocial Outcomes In StrokE (POISE) cohort study. Int. J. Stroke 11, 799–806. doi: 10.1177/1747493016641952 27016514

